# Potential of Diffusion Tensor Imaging and Relaxometry for the Detection of Specific Pathological Alterations in Parkinson's Disease (PD)

**DOI:** 10.1371/journal.pone.0145493

**Published:** 2015-12-29

**Authors:** Regina Esterhammer, Klaus Seppi, Eva Reiter, Bernadette Pinter, Christoph Mueller, Christian Kremser, Tanja Zitzelsberger, Michael Nocker, Christoph Scherfler, Werner Poewe, Michael Schocke

**Affiliations:** 1 Department of Radiology, University Hospital, Innsbruck Medical University, Anichstrasse 35, 6020, Innsbruck, Austria; 2 Department of Neurology, University Hospital, Innsbruck Medical University, Anichstrasse 35, 6020, Innsbruck, Austria; 3 Department of Radiology, University Hospital, Eberhard Karls University, Hoppe-Seyler-Str. 3, 72076, Tübingen, Germany; University of Ulm, GERMANY

## Abstract

The purpose of the present study was to evaluate the potential of multimodal MR imaging including mean diffusivity (MD), fractional anisotropy (FA), relaxation rates R2 and R2* to detect disease specific alterations in Parkinson's Disease (PD). We enrolled 82 PD patients (PD-all) with varying disease durations (≤5 years: PD≤5, n = 43; >5 years: PD>5, n = 39) and 38 matched healthy controls (HC), receiving diffusion tensor imaging as well as R2 and R2* relaxometry calculated from multi-echo T2*-weighted and dual-echo TSE imaging, respectively. ROIs were drawn to delineate caudate nucleus (CN), putamen (PU), globus pallidus (GP) and substantia nigra (SN) on the co-registered maps. The SN was divided in 3 descending levels (SL 1–3). The most significant parameters were used for a flexible discrimination analysis (FDA) in a training collective consisting of 25 randomized subjects from each group in order to predict the classification of remaining subjects. PD-all showed significant increases in MD, R2 and R2* within SN and its subregions as well as in MD and R2* within different basal ganglia regions. Compared to the HC group, the PD≤5 and the PD>5 group showed significant MD increases within the SN and its lower two subregions, while the PD≤5 group exhibited significant increases in R2 and R2* within SN and its subregions, and tended to elevation within the basal ganglia. The PD>5 group had significantly increased MD in PU and GP, whereas the PD≤5 group presented normal MD within the basal ganglia. FDA achieved right classification in 84% of study participants. Micro-structural damage affects primarily the SN of PD patients and in later disease stages the basal ganglia. Iron contents of PU, GP and SN are increased at early disease stages of PD.

## Introduction

Normal findings on neuroimaging were long regarded as a diagnostic prerequisite of classical Parkinson's disease (PD) [1]. Magnetic resonance imaging (MRI) has been mainly employed for the exclusion of other pathologies causing symptomatic parkinsonism like brain tumors or cerebral ischemia [2]. After first reports on T2 signal decreases within the basal ganglia and substantia nigra in PD [3], the quantification of the iron-sensitive T2 and T2* signal has been established by calculating the relaxation times, which is commonly achieved by the read-out of several echos [4]. In MRI, the relaxation times of different weightings characterize the relationship between the spins, meaning the excited nuclei, and their surrounding like other spins and the spin-lattice. The relaxation rates R2 and R2* are the reciprocal values of T2 and T2* relaxation times, respectively, and have been shown to correlate to the iron concentration within the brain tissue, as evaluated in a previous post mortem study [5]. This technique was employed for the assessment of iron deposition in PD and affirmed the prominent iron deposition within the substantia nigra [6–8].

Histological examinations of the underlying pathology in PD indicate that neuronal degeneration spreads slowly from the brain stem to the basal ganglia and even cortical regions [9]. Advanced techniques of diffusion-weighted imaging have been established to visualize and to quantify the movement of water molecules within the brain tissue, providing a mapping of the integrity of the highly developed neuronal architecture [10]. Mean diffusivity (MD) is a stable tissue parameter describing the mobility of water molecules, which is increased in the case of neurodegneration [2]. Fractional anisotropy (FA) is a directional measurement of diffusivity, which ranges from 0 to 1. High FA values reflect movement of water molecules along intact fiber tracts [11]. Both MD and FA enhance the sensitivity of MRI for the detection of micro-structural damages. Reduced FA values have been detected in the lateral part of the substantia nigra permitting complete separation from normal controls in one study [12], which could not be fully reproduced by following studies [13]. diffusion tensor imaging (DTI) studies detected reduced FA values within the substantia nigra, which could not help to differentiate between PD patients and normal controls [14–17]. Recently, reports on increased MD within the substantia nigra of PD patients has arisen [13, 18], which might correspond to micro-structural damages observed in PD by histology.

Several previous studies performed multi-parametric imaging employing as well as relaxation rates for the assessment of pathologic changes in the substantia nigra [14, 15, 19]. However, the focus of these studies was mainly on FA within the substantia nigra, obviously guided by the publication of Vaillancourt and colleagues in 2009 [12]. The purpose of the present study was to evaluate both micro-structural damages and iron load within the substantia nigra and basal ganglia by employing MD, FA, R2 and R2* in order to detect specific alterations in PD at early and late disease stages.

## Materials and Methods

The Ethikkommission of the Medical University Innsbruck approved the present study. All participants gave their written consent, which was stored in the study center.

### Patients and volunteers

The present study was approved by the local ethic committee. Each patient and each volunteer gave informed consent. We enrolled 82 patients with PD, 58 men and 24 women. The median age of the PD patient group (PD-all) was 68.15 years (range: 39–82 years). The median disease duration was 4.3 years (range: 0.2–25 years), the median motor part of the UPDRS (UPDRS-III) 19.5 (range: 6–56), and the median Hoehn & Yahr stages (H&Y) 2 (range: 1–4). The patient group was divided into two subgroups by disease duration. The first group comprised 43 patients (PD≤5) with a disease duration of ≤5 years (median: 2 years; range: 0.2–5 years), a median age of 68 years (range: 38.9–82 years), a median UPDRS-III of 16 (range: 6–42), and a median H&Y of 2 (range: 1–4). The second group contained 39 patients (PD>5) with a disease duration longer than five years (median: 9.5 years; range: 5.2–25 years), a median age of 69.4 years (range: 40–81 years), a median UPDRS-III of 26 (range: 9–56), and a median H&Y of 2.5 (range: 1.5–4).

The healthy control (HC) group comprised 38 subjects without any evidence for neurological or psychiatric diseases, 21 men and 17 women. The median age was 67 years (range: 46–79 years). Since there were no differences in age and gender-distribution between the groups, further analyses were not corrected neither for age nor for gender.

### Magnetic resonance imaging protocol

All measurements were performed on a 1.5 Tesla whole-body MR scanner (Magnetom Avanto, Siemens Erlangen, Germany) with the help of an eight-channel head coil. The imaging protocol comprised a coronal T1-weighted MPRAGE 3D (repetition time (TR), 1600 ms; echotime (TE), 3.44 ms; inversion time (TI), 800 ms; slice thickness (ST), 1.2 mm; matrix, 256 x 224 pixels; number of excitations (NEX), 1; flip angel (FA), 15°; field of view (FoV), 220 x 192.5 mm), a transversal double-echo fast spin echo sequence with T2 and proton density contrast (TR, 3270 ms; TE1-2, 12 and 85 ms; ST, 3–5 mm; spacing between slices (SP), 3.6–6 mm; matrix, 256 x 200; NEX, 1; FA, 150°; FoV, 220 x 171.88 mm; echo train length, 5), a transversal diffusion-weighted echo-planar imaging sequence with diffusion-sensitizing gradients in 12 directions and b-factors of 0 and 1000 s/mm2 (TR, 6000 ms; TE, 94 ms; ST, 3 mm; SP, 3.75 mm; matrix, 128 x 128 (k-space interpolation to 256 x 256); NEX, 1; FA, 90°; FoV, 230 x 230 mm; parallel imaging factor, 2), and a transversal multi-echo T2*-weighted gradient-echo sequence (TR, 200 ms; TE1-8, 2.58 ms, 4.81 ms, 7.04 ms, 9.27 ms, 11.5 ms, 13.73 ms, 15.96 ms, and 18.19 ms; ST, 3 mm; SP, 3.75 mm; matrix 256 x 192 pixels; NEX, 1; FA, 20°; FoV, 230 x 172.5 mm).

### Image analysis

**Fig 1:**
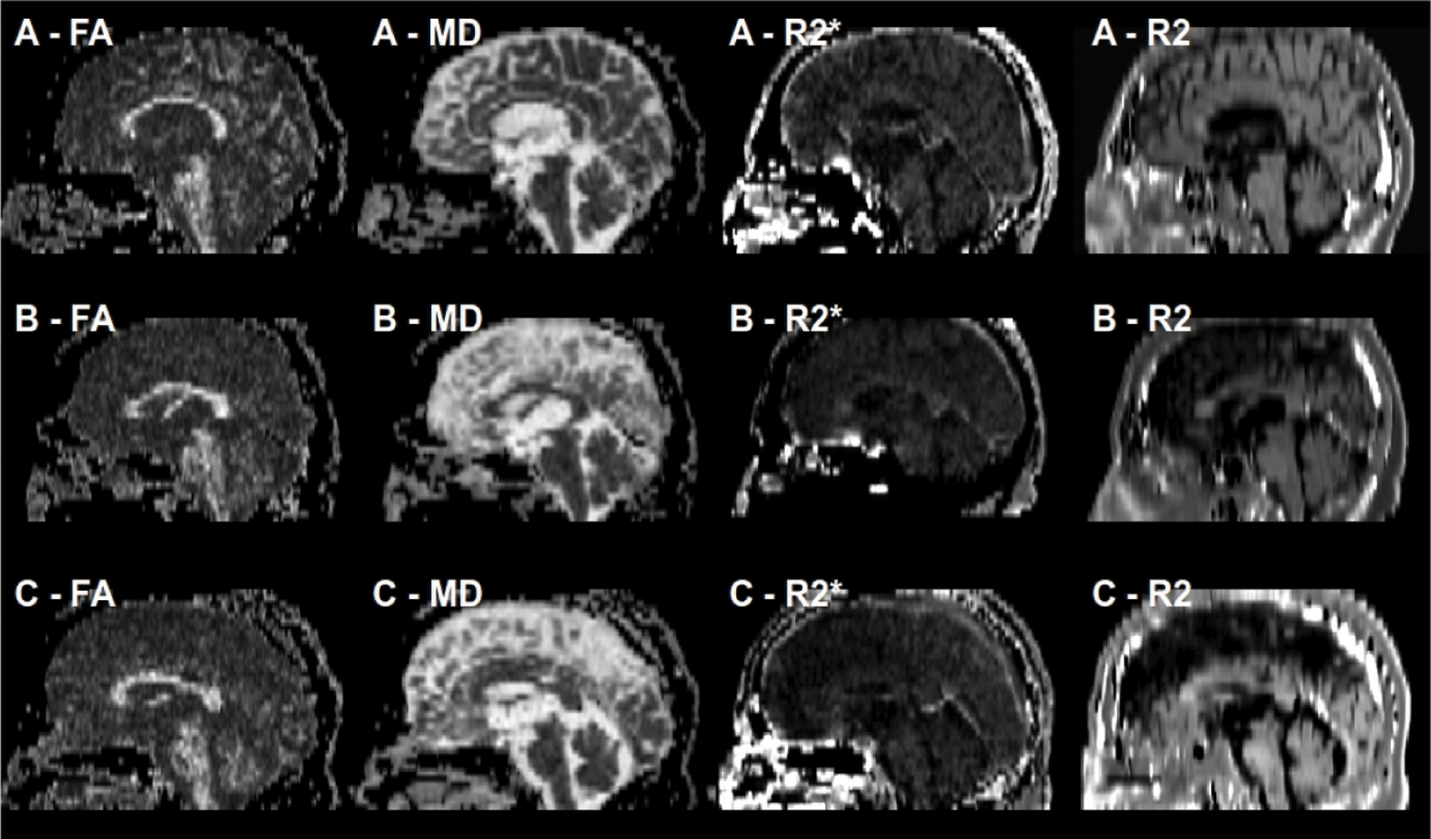
The sagittal reconstructions of the co-registered maps show the excellent coherence in one normal control (A) and in two PD patients (B and C). In four subjects the R2* maps showed distinct signal loss near to the base of the skull as demonstrated in B.

**Fig 2:**
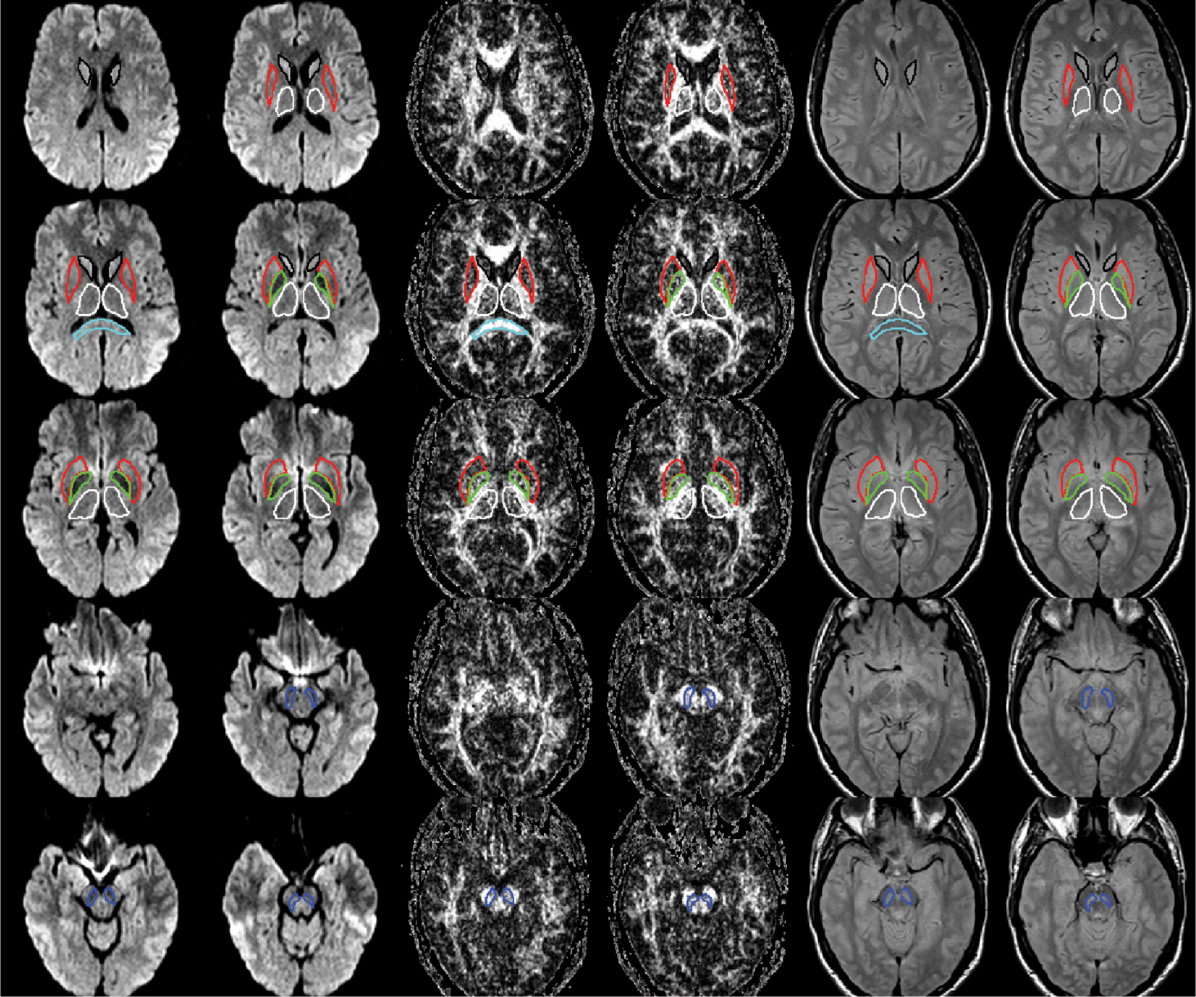
The regions-of-interest (ROIs) were manually drawn by an experienced radiologist by using the b 1000 images, averaged for all measured directions, the FA maps and the proton-density weighted images. For that purpose, the maps of ADC, FA, R2 and R2* as well as the proton-density weighted images were first co-registered. The ROIs were segmented by syncing the b 1000 images, averaged for all measured directions, the FA maps and the proton-density weighted images in ImageJ. The ROIs were stored in the ROI manager and transferred to the co-registered maps. The segmented brain regions were highlighted in different colors: CN—black; PU—red; GP—green; TH—white; SN—blue; CC—cyan.

**Fig 3:**
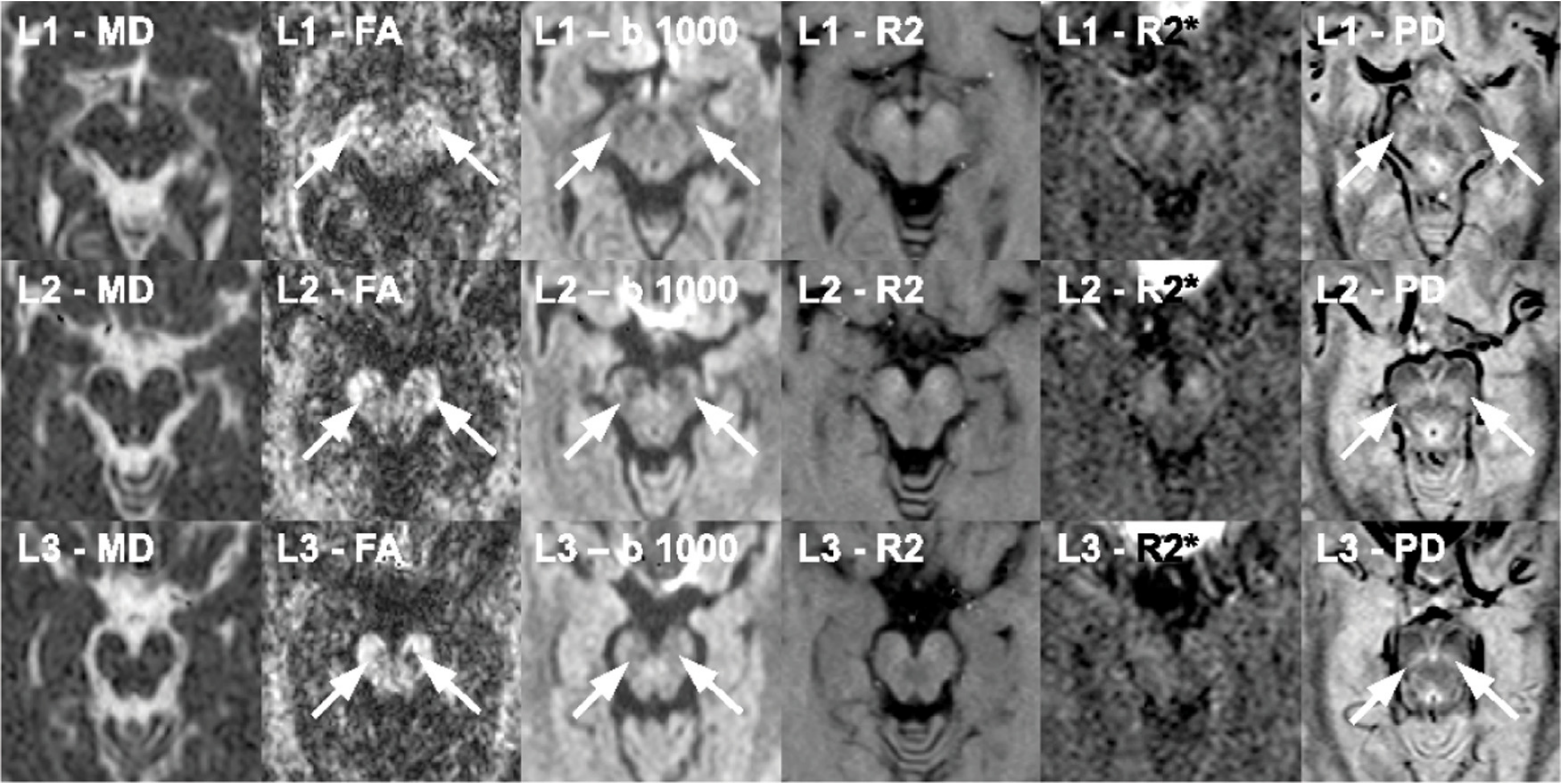
The substantia nigra was evaluated on three levels as shown here in a normal control (male, 61 years old). The delineation of the substantia nigra was poor on the maps of mean diffusivity, given by the mean apparent diffusion coefficient (ADC) in 12 directions as well as on the maps of the relaxation rates R2 and R2*. However, the mean b 1000 images, the FA maps and the proton-density weighted images provided the best contrast for the accurate demarcation of the substantia nigra (SN), which is marked by white arrows. These three different modalities were used for the segmentation of SN.

After transforming the DICOM files into the “nifti” format with the help of the dcm2nii software (Chris Rorden, 64Bit version for Linux), the raw data for diffusion tensor imaging were checked and corrected for motion and eddy current artifacts by using the eddy current correct module of the FDT-FMRIB's toolbox 3.0 as implemented in the FMRIB Software Library (FSL), Release 5.0 [20, 21]. Maps of mean diffusivity (MD) and fractional anisotropy (FA) were calculated by employing the DTIFIT modul of the FDT-FMRIB's toolbox 3.0 [22]. By using the double-echo fast spin echo sequence and the multi-echo gradient-echo sequence, maps of R2 and R2* were determined with the help of an in-house script for ImageJ [23]. Then, the diffusion-weighted images, the R2 and R2*-weighted maps were transferred to the 3D Slicer software in order to co-register the R2 and the R2* maps as well as the MD and the FA maps (Fig 1). For that purpose, we applied the “Pipeline Affine” algorithm of the Expert Automatic Registration tool providing an excellent distortion correction for the MD and the FA maps [24–26]. After co-registration and distortion correction, MD, FA, R2 and R2* maps as well as the diffusion-weighted images were evaluated by using ImageJ, as shown in Fig 2. By using the “Sync Windows” tool (by Joachim Walter at biz.uni-muenchen.de), b1000 images and FA maps were synced for segmentation of different brain regions: putamen (PU), caudate nucleus (CN), globus pallidus (GP), thalamus (TH), splenium of corpus callosum (CC), and substantia nigra (SN). For the segmentation of SN, proton density images were also utilized, as demonstrated in Fig 3. Since previous studies suggested that the caudal portion of the SN manifests the greatest degeneration in PD [7, 27], we considered different levels of the substantia nigra. For this reason, the SN was divided in three descending levels starting at the slice where the red nucleus was visible (SL 1; SL 2; SL 3). CC and TH served as control regions, where we did not expect any alterations. After segmentation, the ROIs were averaged for each brain structure of both sides on the basis of the single pixel values.

#### Statistical evaluation

Statistical evaluation was performed R 2.10.1 [28, 29]. First the data were tested for normal distribution by employing the Shapiro-Wilk test [30]. The evaluation of group differences was performed twice. First, differences in MD, FA, R2 and R2* derived from the different segmented regions between the whole patient collective and healthy controls were assessed with help of the unpaired t-test or the Mann-Whitney-U test in case of normally distributed data or not normally distributed data, respectively. The Type I error rate for the group-wise comparisons (i.e. PD vs. HC) of the different ROIs (i.e. PU, CN, GP, CC, TH, SN, SN-SL1, SN-SL2, SN-SL3) was adjusted by Bonferroni correction using P = 0.05 as global level of significance. Therefore, due to the multiple comparisons of the ROIs, the significance level for the Mann-Whitney-U tests was set at 0.006 (p = 0.05/9).

The second evaluation comprised a subgroup analysis. The patient collective was divided by disease duration(HC, PD≤5, PD>5) as indicated above. Overall significant effects of the subgroup analysis (HC, PD≤5, PD>5) were assessed with the help of the ANOVA for repeated measurements or the Kruskal-Wallis test, depending on data distribution, whereby for the latter the significance level was corrected to 0.006 (P = 0.05/9). The Type I error rate for the post-hoc group-wise comparisons (i.e. PD≤5 vs. HC, PD>5 vs. HC, PD≤5 vs. PD>5) was adjusted by Bonferroni correction using P<0.05 as global level of significance. In case of normal distribution, posthoc t-tests were performed by using following R function:

pairwise.t.test(pd.groups$MD, pd.groups$Disease, p.adj = "bonferroni", paired = FALSE, exact = F)

Otherwise, we employed non-parametric testing by using following R function:

pairwise.wilcox.test(pd.groups$MD, pd.groups$Disease, p.adj = "bonferroni", paired = FALSE, exact = F)

Both methods parametric and non-parametric testing were done in the unpaired mode and supported by automatic group comparison with Bonferroni correction.

The most significant parameters, comprising the MD values of CN, PU, GP, SN, SL1-3, the R2 values of GP, SN, SL1-2, and the R2* values of NC, GP, SN and SL1-3, were subjected to a discriminant analysis by using the linear discriminant analysis LDA for normally distributed data and the flexible discriminant analysis FDA for not normally distributed data as implemented in the “MASS” package and in the “mda” package of R, respectively [31, 32]. For that purpose, 25 subjects of each group were randomized for training sets with the help of the “sample” function in R. The results of LDA/FDA were applied to the remaining test collective.

In addition, correlations between the different MR parameters and clinical parameter as disease duration, the UPDRS-III and the H&Y were evaluated by the Pearson correlation coefficient r for normally distributed data and the Spearman correlation coefficient r for not normally distributed data by using a significance level at <0.001.

## Results

Since MD, FA, R2 and R2* were not normally distributed, non-parametric tests were used for statistical evaluation. The difference in age between the HC and PD-all group was not significant (p = 0.06). The Kruskal-Wallis test revealed a significant effect for age of the different subgroups (HC, PD≤5, PD>5, p<0.001). The post-hoc testing with Bonferroni correction, however did not reveal any significant differences in age between the subgroups (HC vs. PD≤5, p = 1.0; HC vs. PD>5, p = 0.052; PD≤5 vs. PD>5, p = 0.346). Regarding the gender distribution of the HC vs. the PD-all group, the Fisher exact test was not significant (p = 0.10).

### Multimodal imaging with MD, FA, R2 and R2*in different brain regions

#### Comparisons between the entire patient group (PD-all) and healthy controls (HC)

As demonstrated in Tables 1 and 2, the PD-all group showed significant increases in MD within PU as well as within SN, SL 2 and 3 compared to HC. Furthermore, R2* was significantly increased within CN, SL1 and SL3, while R2 was significantly increased within SN, SL1 and SL2 in the PD-all group. The increase in R2* within SN of the PD-all group was just at the limit of significance. We did not detect any significant effects regarding FA. We did not detect any significant effects within the thalamus and corpus callosum.

**Table 1:** Mean diffusivity (MD and fractional anisotropy (FA) are presented as medians for all regions-of-interest (ROIs) of patients with Parkinson's disease (PD-all) and healthy controls (HC). Significant differences were evaluated with help of the Mann-Whitney-U test (M-W, significance level p<0.006). The Kruskal-Wallis test (K-W, significance level p<0.006) was employed for the subgroup analysis comprising HC, PD patients with a disease duration ≤ 5 (PD≤5) and > 5 years (PD>5). Post-hoc testing was done in case of p<0.05. Regarding the K-W tests, the significance level between p<0.05 and p = 0.006 was considered a tendency. Clearly significant effects are marked with bold types.

	HC		PD-all			PD≤5		PD>5		K-W	Bonferroni p-values	
	Median	Range	Median	Range	p-values	Median	Range	Median	Range	p-values	HC vs. PD≤ 5; HC vs. PD>5; PD≤ 5 vs. PD>5	
	MD [x 10^−3^ mm^2^/s]											
Caudate nucleus	0.74	0.66–0.8	0.77	0.66–0.99	0.008	0.75	0.66–0.87	0.77	0.66–0.99	0.009	0.32; 0.008; 0.31	
Putamen	0.72	0.64–0.8	0.75	0.61–0.93	**<0.001**	0.73	0.61–0.89	0.77	0.7–0.93	**<0.001**	1.0; **<0.001**; **<0.001**	
Globus pallidus	0.75	0.63–0.86	0.77	0.6–0.94	0.031	0.76	0.6–0.94	0.79	0.7–0.89	**<0.001**	1.0; **<0.001**; **0.002**	
Thalamus	0.74	0.68–0.81	0.75	0.63–0.86	0.025	0.75	0.66–0.83	0.75	0.63–0.86	0.076		
Corpus callosum	0.76	0.66–0.86	0.77	0.63–0.9	0.153	0.77	0.66–0.9	0.75	0.63–0.9	0.217		
Substantia nigra	0.76	0.69–0.82	0.83	0.69–1.04	**<0.001**	0.8	0.69–0.98	0.84	0.72–1.04	**<0.001**	**<0.001**; **<0.001**; **0.005**	
SN—level 1	0.73	0.65–0.86	0.77	0.64–0.94	0.046	0.76	0.65–0.89	0.78	0.64–0.94	0.036	0.941; 0.035; 0.301	
SN—level 2	0.76	0.68–0.85	0.83	0.64–1.07	**<0.001**	0.82	0.64–1	0.84	0.72–1.07	**<0.001**	**0.006**; **<0.001**; **0.037**	
SN—level 3	0.78	0.61–0.89	0.89	0.71–1.18	**<0.001**	0.87	0.71–1.17	0.9	0.74–1.18	**<0.001**	**<0.001**; **<0.001**; 0.491	
	Fractional anisotropy											
Caudate nucleus	0.35	0.29–0.42	0.34	0.23–0.45	0.151	0.34	0.23–0.43	0.34	0.23–0.45	0.336		
Putamen	0.38	0.34–0.47	0.39	0.28–0.48	0.763	0.4	0.28–0.48	0.38	0.28–0.45	0.134		
Globus pallidus	0.46	0.4–0.55	0.45	0.33–0.57	0.131	0.45	0.33–0.57	0.44	0.34–0.51	0.097		
Thalamus	0.44	0.34–0.5	0.44	0.36–0.51	0.78	0.44	0.36–0.5	0.45	0.37–0.51	0.946		
Corpus callosum	0.8	0.73–0.88	0.81	0.72–0.87	0.552	0.8	0.72–0.86	0.81	0.73–0.87	0.4		
Substantia nigra	0.57	0.5–0.66	0.55	0.41–0.67	0.285	0.54	0.45–0.66	0.56	0.41–0.67	0.347		
SN—level 1	0.56	0.48–0.67	0.56	0.43–0.72	0.683	0.54	0.42–0.7	0.57	0.46–0.72	0.339		
SN—level 2	0.56	0.45–0.66	0.55	0.41–0.7	0.746	0.54	0.41–0.67	0.56	0.42–0.7	0.638		
SN—level 3	0.56	0.45–0.7	0.54	0.35–0.74	0.045	0.54	0.44–0.67	0.54	0.35–0.74	0.133		

**Table 2:** The relaxation rates R2 and R2* are presented as medians for all regions-of-interest (ROIs) of patients with Parkinson's disease (PD-all) and healthy controls (HC). Significant differences were evaluated with help of the Mann-Whitney-U test (M-W, significance level p<0.006). The Kruskal-Wallis test (K-W, significance level p<0.006) was employed for the subgroup analysis comprising HC, PD patients with a disease duration ≤ 5 (PD≤5) and > 5 years (PD>5). Post-hoc testing was done in case of p<0.05. Regarding the K-W tests, the significance level between p<0.05 and p = 0.006 was considered a tendency. Clearly significant effects are marked with bold types.

	HC		PD			PD<5		PD>5		K-W	Bonferroni p-values
	Median	Range	Median	Range	p-values	Median	Range	Median	Range	p-values	HC vs. PD≤ 5; HC vs. PD>5; PD≤ 5 vs. PD>5
	R2 [1/s]										
Caudate nucleus	9.53	8.74–10.68	9.78	7.55–12.21	0.071	9.86	9.02–12.21	9.61	7.55–12.19	0.015	0.011; 1.0; 0.135
Putamen	10.3	9.4–11.3	10.41	8.51–13.52	0.056	10.62	9.4–13.52	10.37	8.51–12.77	0.024	0.018; 1.0; 0.222
Globus pallidus	11.09	9.31–12.81	11.33	9.6–15.84	0.071	11.56	9.98–15.84	10.96	9.6–14.68	0.006	0.006; 1.0; 0.056
Thalamus	9.65	9.04–10.89	9.69	8.9–12.45	0.285	9.72	8.95–12.45	9.58	8.9–12.13	0.206	
Corpus callosum	9.46	8.34–10.73	9.62	7.64–12.58	0.263	9.73	7.67–12.58	9.58	7.64–11.33	0.449	
Substantia nigra	9.95	8.96–11.39	10.51	8.97–13.31	**<0.001**	10.75	8.97–13.31	10.23	9.07–12.03	**<0.001**	**<0.001**; 0.551; **0.006**
SN—level 1	10.17	8.83–11.5	10.87	8.44–13.93	**<0.001**	11.46	8.44–13.93	10.4	9.07–13.31	**<0.001**	**<0.001**; 0.151; **<0.001**
SN—level 2	9.9	9–11.87	10.48	8.99–13.55	**0.001**	10.76	9.5–13.55	10.27	8.99–12.22	**<0.001**	**<0.001**; 0.457; 0.075
SN—level 3	9.69	8.35–10.99	9.79	8.33–12.06	0.204	9.84	8.95–12.06	9.65	8.33–11.7	0.122	
	R2* [1/s]										
Caudate nucleus	15.27	11.86–19	16.22	12.52–25.49	**0.004**	16.46	13.88–25.49	15.86	12.52–21.42	0.013	0.013; 0.104; 1.0
Putamen	16.84	13.61–22.12	17.66	13.38–27.68	0.033	18.45	14.96–27.68	17.22	13.38–24.31	0.031	0.024; 0.962; 0.394
Globus pallidus	21.45	17.14–26.83	21.71	15.39–35.49	0.087	22.54	18.19–35.49	21.16	15.39–35.2	0.008	0.016; 1.0; 0.035
Thalamus	16.12	13.84–18.89	16.23	13.42–21.58	0.158	16.15	14.63–21.58	16.34	13.42–21.58	0.351	
Corpus callosum	19.62	17.21–22.11	19.6	16.41–23.1	0.608	19.52	16.81–22.69	19.62	16.41–23.1	0.422	
Substantia nigra	20.58	17.43–23.94	21.25	15.75–29.36	**0.006**	22.43	18.42–27.81	20.85	15.75–29.36	**<0.001**	**<0.001**; 1.0; **0.002**
SN—level 1	20.86	17.81–25.37	22.45	15.57–30.41	**<0.001**	23.4	18.89–27.94	21.61	15.57–30.41	**<0.001**	**<0.001**; 0.903; **<0.001**
SN—level 2	21.58	15.36–25.07	21.15	15.9–32.92	0.356	23.04	18.42–32.92	20.91	15.9–32.76	0.009	0.051; 1.0; 0.021
SN—level 3	19.11	14.81–23.19	20.02	15.32–28.81	**0.002**	20.38	16.99–28.81	19.6	15.32–26.95	**0.002**	**0.001**; 1.0; 0.066

#### Subgroup analyses comparing PD≤5, PD>5 and HC

When comparing all groups with the help of the Kruskal-Wallis test and a corrected significance level (p < 0.006), we revealed significant overall effects for MD within different basal ganglia regions as well as within SN and its subregions, as demonstrated in Tables 1 and 2 (S1 Data). For R2 and R2*, overall significant effects were observed only within SN and its subregions, whereby the overall effects within the basal ganglia were just above the corrected significance level. We did not detect any significant effects for FA. Furthermore, we did not reveal any significant effects within the thalamus and the corpus callosum.

#### Basal ganglia

The PD>5 group exhibited significant increases in MD within PU and GP compared to both HC and the PD≤5 group, while we did not observe any significant differences between the PD≤5 group and HC. In the PD≤5 group, R2 and R2* were increased within all basal ganglia regions group compared to HC, whereby the differences were just above the significance level. We did not observe any significant differences in R2 and R2* between the PD>5 group and HC.

#### Substantia nigra

**Fig 4:**
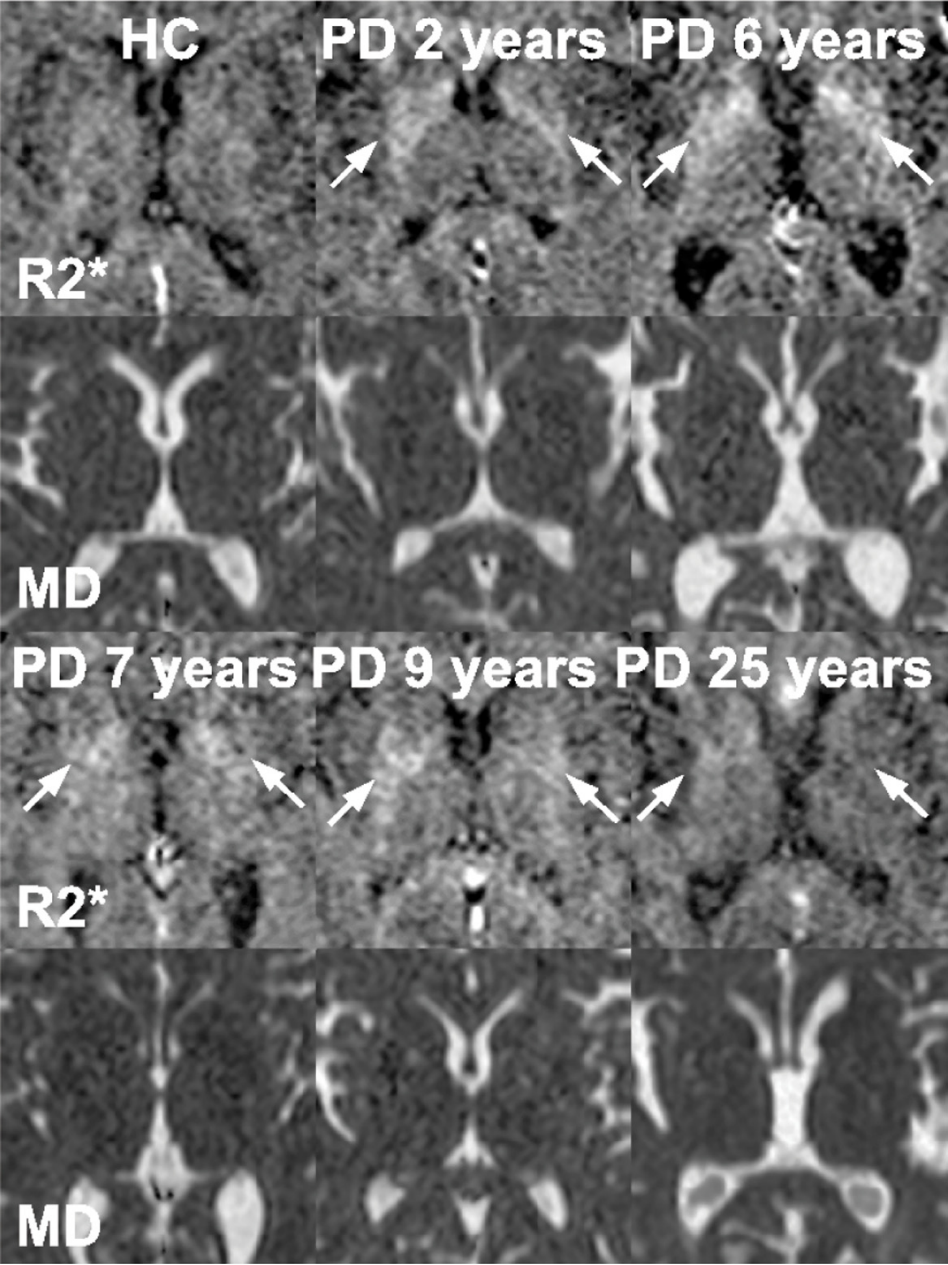
The R2* and the MD maps of the basal ganglia indicate relatively high iron concentrations in patients at early stages of Parkinson's disease (PD). The disease durations are marked on the upper margin of the R2* maps. The corresponding MD maps are just below the R2* maps. The high signals on the R2* maps within the basal ganglia indicate high iron concentrations and pointed out by white arrows. PD patients with longer disease durations show less signal on R2* maps compared to PD patients at early disease stage. Differences in MD can be hardly detected visually, but with the help of a ROI analysis.

**Fig 5:**
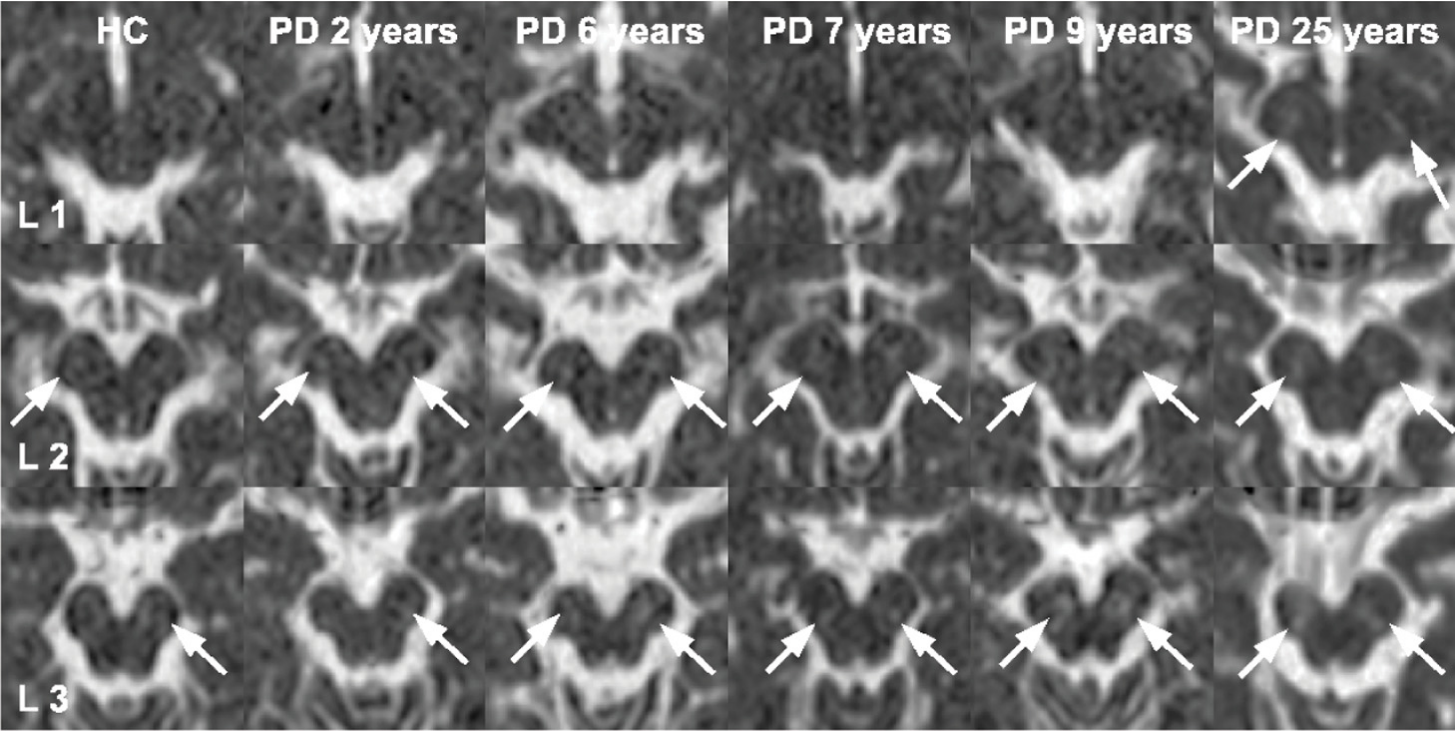
The MD maps are derived from the substantia nigra of a representative healthy control and several PD patients with increasing disease duration as given on the upper margin of each image. The white arrows point out increased signals on MD maps. With increasing disease duration the areas of elevated MD grow, which is also supported by a significant correlation.

**Fig 6:**
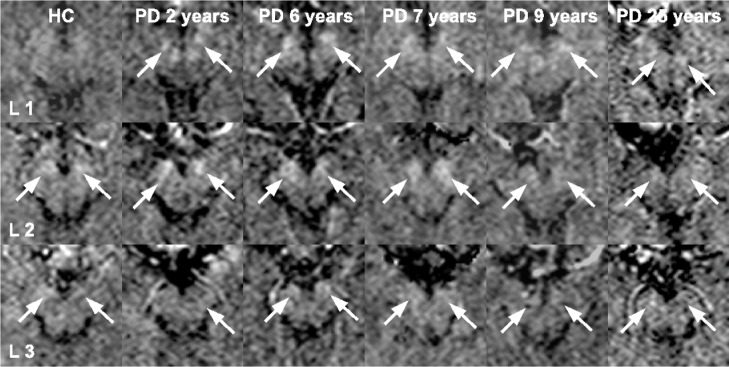
The R2* maps are derived from the substantia nigra of a representative healthy control and several PD patients with increasing disease duration as given on the upper margin of each image. Images belong to the same subjects included in Fig 5. The white arrows point out increased signals on R2* maps, indicating iron overload. With increasing disease duration the areas of elevated R2* become inhomogeneous.

Representative slices of the substantia nigra (SN) are demonstrated in Figs 3–6. Regarding the whole SN, both the PD≤5 and the PD>5 group showed significantly increased MD compared to the HC group, whereby the PD>5 group had significantly higher MD than the PD≤5 group. The PD≤5 group exhibited significantly higher R2 and R2* values than the PD>5 and the HC group.

In level SL1, we did not observe any significant differences in MD. The PD≤5 group exhibited significantly higher R2 and R2* values than the PD>5 and the HC group.

In level SL2, the PD>5 group showed significantly increased MD compared to the HC group. The PD≤5 group showed also increased MD compared to the HC group, whereby the difference was just above the significance level. Furthermore, the PD≤5 group had significantly increased R2 values compared to the HC group.

In level SL3, both the PD≤5 and the PD>5 group showed significantly increased MD compared to the HC group. In addition, the PD≤5 group had significantly higher R2* values than the HC group.

### Multivariate Flexible Discriminant Analysis for between-group discrimination

**Fig 7:**
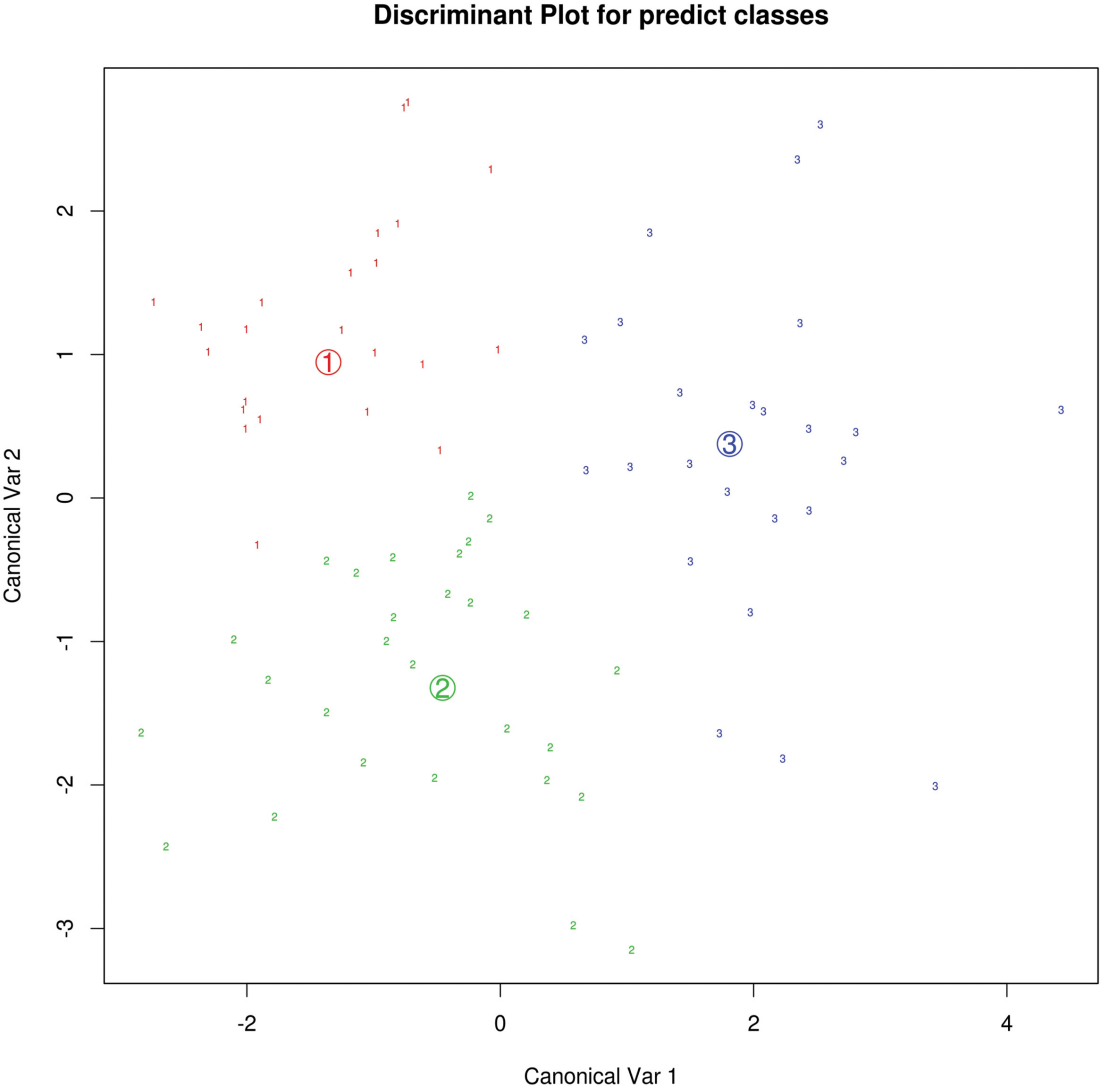
The scatter plot shows the results of the flexible discriminant analysis (FDA) in the training collective consisting of 25 subjects from each group (1, HC; 2, PD≤5; 3, PD>5). The FDA bases on the most significant MR parameters including MD of NC, PU, GP, SN and SL 1–3 as well as R2 of GP, SN, SL 1 and SL 2 as well as R2* of GP, SN, SL 1 SL 2 and SL 3. The misclassification error is 0.13.

25 subjects of each group were randomized to the training set. The most significant parameters were empirically employed for the flexible discriminant analysis (FDA). Including MD of CN, PU, GP, SN and SL 1–3 as well as R2 of GP, SN, SL 1 and SL 2 as well as R2* of GP, SN, SL 1 SL 2 and SL 3 (Fig 7), the training misclassification error was 0.13. When applying the predict classes to the test collective, 11 subjects of HC were correctly classified, while 2 subjects were allocated to the PD≤5 group. Regarding the PD≤5 group, correct classification was achieved in 15 of 18 patients, whereas 3 patients were allocated to the PD>5 group. In the PD>5 group, 12 of 14 patients were correctly classified, 2 patients were allocated to the PD≤5 group. The error of classification was 0.16. Consequently, correct classification was obtained in about 84% of all included subjects.

### Correlation analyses

#### Correlations with disease duration

As demonstrated in Table 3, disease duration showed highly significant correlations with MD values in PU (r = 0.58; p<0.001), GP (r = 0.34; p<0.001), SN (r = 0.41; p<0.001), SL 2 (r = 0.33; p<0.001) and SL 3 (r = 0.25; p<0.001) as well as with R2* in SL 1 (r = -0.36; p<0.001). By contrast, disease duration significantly correlated inversely with R2 in SN (r = -0.26; p<0.001) and SL 1 (r = -0.38; p<0.001).

**Table 3:** The Spearman correlation coefficients r were determined to evaluate the relationship between the MR parameters (MD, mean diffusivity; FA, fractional anisotropy, R2, relaxation rate R2; R2*, relaxation rate R2*) and the clinical data. Significant correlations (p<0.001) were presented in bold types.

	MD	FA	R2	R2*
Region	Spearman r	Spearman r	Spearman r	Spearman r
Caudate nucleus				
Hoehn & Yahr	0.14	0.12	-0.02	0.03
UPDRS 3	0.23	-0.04	0.1	0.23
Disease duration	0.2	0.06	-0.16	-0.01
Putamen				
Hoehn & Yahr	0.22	0.17	0.03	-0.02
UPDRS 3	0.29	-0.05	0.12	0.15
Disease duration	**0.58**	0.02	-0.12	-0.08
Globus pallidus				
Hoehn & Yahr	0.24	0	-0.15	-0.25
UPDRS 3	**0.34**	-0.1	-0.12	-0.18
Disease duration	**0.34**	-0.02	-0.22	-0.27
Substantia nigra				
Hoehn & Yahr	0.1	0.17	-0.13	-0.18
UPDRS 3	0.24	-0.18	-0.12	-0.19
Disease duration	**0.41**	-0.31	-0.26	-0.31
Substantia nigra L1				
Hoehn & Yahr	0.06	0.23	-0.21	-0.17
UPDRS 3	0.18	-0.07	-0.21	-0.25
Disease duration	0.2	-0.2	**-0.38**	**-0.36**
Substantia nigra L2				
Hoehn & Yahr	0.11	0.07	-0.04	-0.1
UPDRS 3	0.2	-0.3	-0.07	-0.11
Disease duration	**0.33**	-0.41	-0.18	-0.24
Substantia nigra L3				
Hoehn & Yahr	0.1	0.13	-0.07	-0.13
UPDRS 3	0.18	-0.16	-0.11	-0.07
Disease duration	**0.25**	-0.24	-0.16	-0.22

#### Correlations to UPDRS-III

UPDRS-III correlated significantly with MD within GP (r = 0.34; p<0.001).

#### Correlations to Hoehn & Yahr stages

H&Y did not show any significant correlations.

## Discussion

In the present study, we performed multi-modal imaging in 82 PD patients at early and late disease stages including MD, FA as well as the relaxation rates R2 and R2* on a 1.5 Tesla MR scanner to detect disease specific alterations in the SN and basal ganglia. Compared to the HC group, the entire PD group showed significant increases in MD, R2 and R2* within the SN and its subregions as well as increases in MD within PU and R2* within CN. However, our findings became differentiated, when dividing our patient group by disease duration. Both the PD≤5 group and the PD>5 group showed increased MD within SN, especially at the basal levels of SN, while the PD>5 group had significantly elevated MD within PU and GP. Accordingly, MD within SN and its subregions as well as within PU and GP correlated significantly with disease duration. R2 and R2* values were significantly increased within the SN and most of its subregions tended to a significant elevation within GP and the remaining basal ganglia regions. A FDA, including the most significant parameters, achieved right classification in 84% of study participants.

Despite the fact that 3 Tesla MR scanners are increasingly used for imaging and research, the working horse for routine is still the 1.5 Tesla system. The advantage of a 1.5 Tesla MR scanner is the more homogeneous magnetic field compared to higher B0 amplitudes. Consequently the efforts for image correction and filtering are less. On the other hand, MR imaging means four times as much signal-to-noise compared to 1.5 Tesla, which can be used for a better resolution and image quality [33]. The most important finding of the present study is the diversification of the results, when dividing the entire PD patient group into two subgroups by disease duration. The median disease duration of our entire patient collective was 4.3 year and, therefore, fully comparable to the previous studies mentioned above, whereby we had a substantial portion of PD patients with a relatively long disease duration up to 25 years. Since we suspected that some MR parameters might be related to disease duration, we divided the patient group by disease duration, whereby the cut-off of five years was arbitrarily chosen. Up to now, imaging studies focusing on disease duration in PD are very rare [34].

When regarding the entire patient group, our results appears to be fully comparable to the results of several previous studies. We obtained significantly increased R2 and R2* values within SN and its subregions, commonly considered markers of iron overload [5] and previously reported by several studies [7, 8, 14, 15, 35, 36]. PD patients showed also slightly decreased FA within SL3, the lowest level of SN, as also suggested by previous studies at 1.5 [16, 17] and 3 Tesla [12, 14, 15]. Furthermore, we detected increased MD within SN and its subregions, which was recently described by two different publications [13, 18]. Some features obtained by the present study were rarely or not previously observed, comprising increased R2* within CN and PU [37] as well as increased MD within CN, PU and GP [38]. Increased MD commonly reflects increased extracellular space due to neuronal degeneration and consecutive gliosis [39]. In principle, this effect is known from atypical parkisonian disorders like multiple system atrophy or progressive supranuclear palsy, whereby those disease groups exhibit much higher MD values within the basal ganglia than the PD>5 group according to the disease progression rate [40–43].

Evaluating the two PD subgroups in comparison to the HC group, both the PD≤5 and PD>5 group showed increased MD within SN, SL2 and SL3, whereby the PD>5 group exhibited significantly higher MD values in SN and SL2 than the PD≤5 group. However, PD≤5 group exhibited normal MD values within the basal ganglia, while the PD>5 group had significantly elevated MD values within PU and GP, and tended to increased MD values within CN. First, our results support a prominent involvement of SN into neuronal degeneration in PD. Accordingly, recent high-field MR studies have suggested that the majority of PD patients is affected by degeneration of nigrosome 1, which was diagnosed as lacking of a hyperintense structure within SN iron-sensitive images [44–46]. Furthermore, our findings indicate that PD patients suffer from ascending neuronal degeneration with disease duration. Corresponding to the differences in regional MD between both patient groups, we detected significant correlations between MD and disease duration within all subregions of SN as well as within PU and GP. Moderate correlations between MD and UPDRS-III were observed within the basal ganglia and SN. From the pathological point of view, our findings fit with the currently established hypothesis for the pathogenesis of PD. The pathology is supposed to start in the dorsal motor nucleus of the vagal nerve as a upward-moving process, spreading out in the reticular formation. Subsequently, the degeneration crosses the upper limit of the pontine tegmentum and enters the basal portions of the midbrain, involving the pars compacta of SN [47]. These regions are affected by neuronal loss, probably triggered by oxidative stress and neuroinflammation [48]. In later disease stages, the PD pathology spreads to the basal ganglia and cerebral cortex [47].

The subgroup analysis of R2 and R2* revealed some unexpected results. PD patients with a short disease duration exhibited not only increased R2 and R2* within SN and its subregions, as observed in the entire patient collective, but also within the basal ganglia compared to the HC group, whereby these increases were just above the corrected significance level. In contrast, PD patients with longer disease duration did not show any significant differences in R2 and R2* compared to HC. A comparison to the literature is difficult, because patient collectives of previous studies were not divided by disease duration, whereby the majority of previous studies detected increased relaxation rates only within the SN [7, 8, 14, 15, 35, 36]. Only a few previous MR studies reported on increase in relaxation rates within the basal ganglia of PD patients [3, 37]. Only one previous study reported on longitudinal, slight increases in R2* within the SN and caudal CN over a period of three years [34]. However, longitudinal imaging studies in PD are difficult because of the very slow disease progression [49]. Our explanation for the lack of significantly increased relaxation rates in the PD>5 group focuses on the different processes occurring within the same ROI, iron accumulation and neuronal degeneration. While iron accumulation leads to an increase in R2 and R2* signal [5], neuronal degeneration and consecutive gliosis counteract this effect by lengthening T2 relaxation times within the tissue [50]. Both processes within the same ROI may result in different and inhomogeneous signal intensities as known from various signal intensities in multiple system atrophy [51] and from the eye of the tiger sign [52] and might explain our R2* and R2 signals in the PD>5 group. From the histological point of view, iron seems to interact with α-synuclein forming the Lewy bodies [53], which spread from the midbrain to the striatum with increase in disease duration. This spreading of Lewy bodies might be an indicator for an iron release within the striatum, probably triggering neuronal death with consecutive gliosis [54, 55].

Another interesting topic discussed in the recent literature is the involvement of FA within SN. Early DTI studies performed on 1.5 Tesla machines indicated reduced FA values in the basal portions of SN [16, 17]. Accordingly, we detected small but non-significant FA decreases in SL 3 of our entire PD patient collective. However, the subgroup analysis of the PD patients separated by disease duration did not reveal any significant effects. Several recent studies carried out on 3 Tesla MR scanners observed more pronounced reductions of FA within SN in PD [12, 14, 15]. One of these studies yielded a complete separation of PD patients and normal controls based on reduced FA values [12]. From the technical point of view, FA is a measure of anisotropy due to the architecture of myelinated fibers and is sensitive to white matter diseases [56]. The importance of FA changes is questionable. Furthermore, some previous studies discussed on hints that iron overload might lead to an increase in FA [57–59]. Thereby, the interpretation of FA changes within gray matter seems to be complex.

Since our findings partly bases on only small, but significant changes, such as those observed for the MD values within the basal ganglia, which reveal themselves only through the ROI analysis, we employed a discrimination analysis. The intention of the discrimination analysis was to confirm that our results, which appeared to be very heterogeneous and partly contradictive to the literature, can be classified in specific patterns and therefore used for further into the pathology of PD. For that purpose, we employed the flexible discriminant analysis (FDA), including the most significant MR parameters, because our data were not normally distributed. The FDA analysis yielded an excellent separation of the different patient subgroups with a low error rate, after determining the predict classes with the help of randomized training collective. This result supports our observation that PD patients with a shorter disease duration below five years and those with a longer disease duration above five years have different imaging patterns, probably caused by the sequela of micro-structural damages, as expected from the Parkinson's pathology. As we have included a lot of MR parameters to the FDA analysis, the value for clinical routine imaging might be limited at the moment. Our findings might help to understand the disease progression.

In early disease stage of PD, iron is significantly increased within SN and tendencially elevated within the basal ganglia, as already detected in lot of other neurodegenative disorders like atypical parkinsonian disorders, Alzheimer's disease, multiple slcerosis, amyotrophic lateral sclerosis, neurodegeneration with brain iron accumulation or Friedreich's ataxia [60]. It is still unknown, whether iron accumulation within the brain tissue is the cause or the consequence of degeneration [61]. Numerous previous studies have confirmed that SN is affected by iron accumulation in PD, some reports have also proved that iron also occur within the basal ganglia in PD [60]. However changes in iron over time were controversially detected and discussed [60]. Furthermore, it is known that that iron is closely associated with neuromelanin, which is located in neurons containing dopamine. The loss of these neurons is believed be a key moment in the pathology of PD [62]. As already discussed above, the interaction between neuromelanin and α-synuclein could be associated with iron release triggering inflammation, neuronal loss and gliosis, whereby these processes proceed to the basal ganglia. As shown by the FDA analysis, these pathological changes might be the substrate for the different imaging patterns depending on disease duration.

In conclusion, our data suggest different imaging patterns in PD patients with disease durations below and above five years. In both patient groups, significant increases in MD were only observed in the lower and the middle level of SN compared to the HC group. However the PD>5 group presented significantly higher MD values in the middle level than the PD≤5 group, whereby the PD≤5 group exhibited significantly increased R2 and R2* within SN and its subregions, and tended to increased R2 and R2* within the basal ganglia regions. Exactly at the latter location, PD patients at later disease stages suffered from increased MD, suggesting that iron accumulation in PD precedes neuronal degeneration. Due to our approach to segment entire anatomical structures, not only containing the iron-bearing parts of SN and basal ganglia, advanced micro-structural damages as indicated by increased MD might counteract R2* and R2 increases due to iron. Including the most significant parameters, the flexible discriminant analysis obtained an excellent separation of the patient subgroups supporting the observation of different imaging patterns and pointing out that the disease duration should be addressed in PD imaging studies. Although the excellent result of the flexible discriminant analysis was based on a lot of MR parameters, we believe that a combined MR protocol comprising DTI and relaxometry could serve as a feasible MR protocol for clinical routine. Further studies should be carried out, in order to investigate this issue.

## Supporting Information

S1 Data The values of the ROI analysis are available as a supporting information file, containing the ROI values of MD, FA, R2 and R2* for each patient and for each volunteer. (CSV)
